# A protocol for recombinant protein quantification by densitometry

**DOI:** 10.1002/mbo3.1027

**Published:** 2020-04-07

**Authors:** Susana María Alonso Villela, Hazar Kraïem, Balkiss Bouhaouala‐Zahar, Carine Bideaux, César Arturo Aceves Lara, Luc Fillaudeau

**Affiliations:** ^1^ TBI CNRS INRAE INSA Université de Toulouse Toulouse France; ^2^ Laboratoire des Venins et Molécules Thérapeutiques Institut Pasteur de Tunis Université Tunis El Manar Tunis Tunisia; ^3^ Faculté de Médecine de Tunis Université Tunis El Manar Tunis Tunisia

**Keywords:** densitometry, image analysis, nanobody production, protein quantitation, SDS‐PAGE

## Abstract

The protein purity is generally checked using SDS‐PAGE, where densitometry could be used to quantify the protein bands. In literature, few studies have been reported using image analysis for the quantification of protein in SDS‐PAGE: that is, imaged with Stain‐Free™ technology. This study presents a protocol of image analysis for electrophoresis gels that allows the quantification of unknown proteins using the molecular weight markers as protein standards. *Escherichia coli* WK6/pHEN6 encoding the bispecific nanobody CH10‐12 engineered by the Pasteur Institute of Tunisia was cultured in a bioreactor and induced with isopropyl β‐D‐1‐thiogalactopyranoside (IPTG) at 28°C for 12 hr. Periplasmic proteins extracted by osmotic shock were purified by immobilized metal affinity chromatography (IMAC). Images of the SDS‐PAGE gels were analyzed using ImageJ, and the lane profiles were obtained in grayscale and uncalibrated optical density. Protein load and peak area were linearly correlated, and optimal image processing was then performed by background subtraction using the rolling ball algorithm with radius size 250 pixels. No brightness and contrast adjustment was applied. The production of the nanobody CH10‐12 was obtained through a fed‐batch strategy and quantified using the band of 50 kDa in the marker as reference for 750 ng of recombinant protein. The molecular weight marker was used as a sole protein standard for protein quantification in SDS‐PAGE gel images.

## Graphical abstract

This article proposes a protocol for image analysis of electrophoresis gels that allows the quantification of unknown proteins using the molecular weight markers in SDS‐PAGE gels as protein standards. The periplasmic production of the nanobody CH10‐12 was obtained through fed‐batch strategy and quantified using the band of 50 kDa in the marker as a reference for 750 ng of recombinant protein. The molecular weight marker was used as a sole protein standard for protein quantification in SDS‐PAGE gel images.

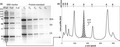

## 1 INTRODUCTION

*Escherichia coli* is a well‐known microorganism used as a workhorse in the production of recombinant therapeutic molecules, such as antibodies and antibody fragments (Graumann & Premstaller, 2006). Lately, nanobodies, small fragments of camelid antibodies, have been proven to neutralize scorpion toxins (Alirahimi et al., 2018; Hmila et al., 2008). The periplasmic production of these nanobodies has been studied in shake flasks induced by synthetic IPTG under the control of the *lac* promoter (Laustsen et al., 2016).

The nanobodies are normally obtained after purification of periplasmic proteins using IMAC (Pardon et al., 2014). The identification and quantification of these nanobodies are of the utmost importance during the purification steps. SDS‐PAGE is used to check and characterize the purified recombinant proteins, and colorimetric and ultraviolet absorption methods are used to quantify them (Bradford, 1976; Stoscheck, 1990).

The semiquantification of protein load in the gel is made through a nonstandardized methodology, using densitometry in SDS‐PAGE and Western blot assays (Gassmann, Grenacher, Rohde, & Vogel, 2009). The use of external protein standards of known concentration is commonly used for the quantification of the electrophoresis‐based separated samples (Holzmüller & Kulozik, 2016; Rehbein & Schwalbe, 2015; Vincent, Cunningham, Stephens, Halayko, & Fisher, 1997). Stain‐Free^TM^ technology by Bio‐rad (USA) has been applied by Holzmüller and Kulozik (2016) and Gürtler et al. (2013), in which the ultraviolet fluorescence of the protein of interest was used as the quantifying parameter of the unknown samples.

Image analysis by densitometry is made using the profiles of the lanes and calibrated to a known standard (Cromey, 2010; Syrový & Hodný, 1991). The peak area is the signal used in most densitometry analysis (Gassmann et al., 2009; Gorr & Vogel, 2015; Rehbein & Schwalbe, 2015), and the peak maximum intensity has been utilized in the quantification of proteins in Western blots imaged by fluorescence (Gürtler et al., 2013; Holzmüller & Kulozik, 2016). The volume of the peak is not commonly considered (Vincent et al., 1997) since the correlations obtained often yield inaccurate protein load estimation (Gassmann et al., 2009).

In the present work, a new protocol of image analysis for electrophoresis gels is reported. The protocol describes the quantification of protein in the bands of SDS‐PAGE gels. The unknown proteins are quantified with commercially available protein standards: bovine serum albumin (BSA), carbonic anhydrase (CA), and ovalbumin (OV), and molecular weight markers of known concentration. The molecular weight marker is then used as a sole protein standard for protein quantification and molecular weight estimation. The protocol was applied to the quantification of the periplasmic production of a recombinant nanobody that neutralizes specific toxins in the scorpion venom.

## 2 MATERIALS AND METHODS

### 2.1 Nanobody protein

Experiments were conducted with *Escherichia coli* K12/WK6 {∆(lac‐pro), galE, strA, nal; F’ lacI^q^ Z∆M15, pro+} harboring pHEN6 plasmid (derived from pBR322) encoding the chimeric format of the bispecific nanobody VHH10‐VHHF12 (called CH10‐12), retrieved from the combinatorial libraries. The strain was engineered by the Pasteur Institute of Tunisia (Kraiem, 2018). The nanobody CH10‐12 has an estimated molecular weight of 31 kDa, and it neutralizes the toxins present in the groups AahI’ and AahII of the *Androctonus australis hector* scorpion venom (Hmila et al., 2010).

**Figure 1:**
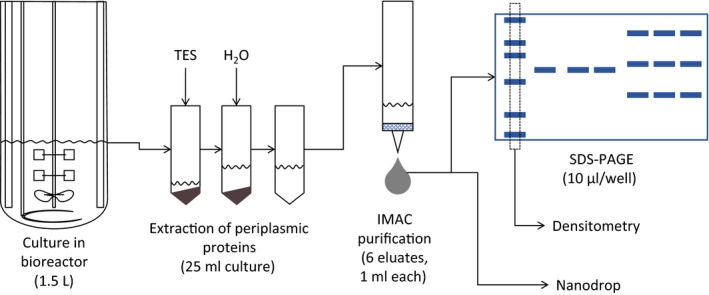
Experimental setup and purification steps of the nanobody CH10‐12

Cultures were performed in a 5 L bioreactor (Figure 1), Biostat B‐DCU (Sartorius) using glucose as a carbon source in 1.5 L of minimal mineral culture medium (Sunya, Delvigne, Uribelarrea, Molina‐Jouve, & Gorret, 2012). The batch phase was conducted at 37°C, and at the depletion of the 10 g/L of initial glucose, a fed‐batch mode was applied with an exponential feed of a glucose solution at 300 g/L, imposing a specific growth rate of µ = 0.38 per hour.

Residual glucose and organic acids were quantified by HPLC (Aminex column HPX‐87H, Bio‐rad). Biomass cell dry weight was determined by a gravimetric method using preweighted microcentrifuge tubes (Eppendorf).

Protein expression was induced at 23 g cdw/L with 1 mM of isopropyl β‐d‐1‐thiogalactopyranoside (IPTG, Sigma‐Aldrich) at 28°C for 12 hr. At induction, the glucose feed rate was set to 4.5 g/hr of glucose imposing a µ ≤ 0.03 per hour.

Samples of 25 ml of the cell suspension were taken every 2 hr, and cells were harvested by centrifugation at 8,228 g and 4°C for 8 min. Cells were suspended in 1.8 ml ice‐cold TES buffer (200 mM Tris pH 8.0, 0.5 mM EDTA pH 8.0, 0.5 M Sucrose) and incubated at 4°C for 2 hr under agitation at 350 rpm in a Thermomixer comfort (Eppendorf). The osmotic shock was performed adding 3.2 ml of cold water to the cell suspension and incubated at 4°C for 2 hr under agitation at 350 rpm in a Thermomixer comfort (Eppendorf; Neu & Heppel, 1965). After the addition of 46 µl of MgCl_2_ 2 M (Sigma‐Aldrich), the cell suspension was centrifuged at 8,228 g and 4°C for 30 min and the periplasmic extract was recovered (Pardon et al., 2014).

Periplasmic proteins were purified by immobilized metal affinity chromatography (IMAC) using His‐Select Nickel Affinity Gel (Sigma‐Aldrich). The nanobody protein was eluted from the column with phosphate‐buffered saline (PBS) and 250 mM Imidazole (Sigma‐Aldrich), pH 7.54. Six elute fractions, of 1 ml each, were obtained and stored at 4°C (Figure 1).

### 2.2 Protein standards

Three proteins were used as standards: Bovine serum albumin (BSA, Sigma‐Aldrich) lyophilized powder for gel electrophoresis with a molecular weight of 66 kDa; albumin from chicken egg white or ovalbumin (OV, Sigma‐Aldrich) lyophilized powder for gel electrophoresis with a molecular weight of 45 kDa, and carbonic anhydrase from bovine erythrocytes (CA, Sigma‐Aldrich) lyophilized powder for enzyme analysis with a molecular weight of 30 kDa.

The BSA is a cheap, well‐known protein commonly used in protein quantification (Bradford, 1976). The ovalbumin (OV) and the carbonic anhydrase (CA) have a molecular weight close to that of the protein of interest.

A standard of each protein was prepared. The absorbance of each solution was measured in a Nanodrop spectrophotometer 1,000 (Thermo Fisher Scientific) at a wavelength of 280 nm. The Beer–Lambert law was used for the calculation of the final protein concentration of each solution using the percent extinction coefficient (*ε*_1%_) of each protein for a wavelength of 280 nm (Equation 1).

(1) $$C=\frac{A_{280}}{\varepsilon_{1\%}l}$$

Concentrations for BSA, CA, and OV were 8.9, 9.1, and 5.9 mg/ml respectively. A mixture of the proteins was prepared by combining 100 µl of each protein standard in a microcentrifuge tube (Eppendorf) for a final concentration of 3 mg/ml for BSA and CA, and 2 mg/ml for OV. Successive dilutions were made from this protein mixture, and five concentrations were prepared, of approximately, 0.01, 0.03, 0.06, 0.1, and 0.3 mg/ml per protein mixture. All protein standards were stored in aliquots at −20°C until use.

For the SDS‐PAGE, the ready‐to‐use Precision Plus Protein Unstained Protein Standards (Bio‐rad) were used as molecular weight markers. The marker contains ten recombinant protein bands of 250, 150, 100, 75, 50, 37, 25, 20, 15, and 10 kDa.

According to the manufacturer, the protein bands of 75, 50, and 25 kDa are reference markers within the molecular weight marker, as they have three times the intensity of the other bands. Similarly, for a 10 µl lane of molecular weight marker, the band of 50 kDa has 750 ng of protein, and the bands of 20 and 100 kDa have 150 ng of protein, each. The nature of the recombinant proteins in the molecular weight marker is not described by the manufacturer.

### 2.3 SDS‐PAGE

Protein samples were diluted at a ratio 1:1 with Laemmli 2× buffer solution (Bio‐rad) with 5% 2‐mercaptoethanol (Sigma‐Aldrich) as a denaturing agent and heated in a water bath at 90°C for 10 min.

**Figure 2:**
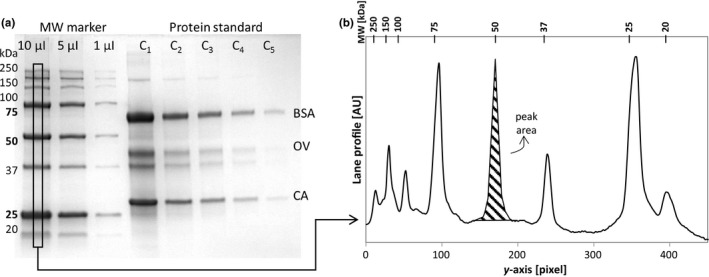
Qualitative analysis made on the gels. (a) Image of the SDS‐PAGE used for the tests. (b) Lane profile and peak area determination

Protein samples of 10 µl were poured in the wells of an Any kD Mini‐Protean TGX Stain‐Free™ Precast gel (Bio‐rad). The molecular weight marker was loaded in three wells at 10, 5, and 1 µl without dilution in Laemmli buffer (Figure 2a).

Electrophoresis was run at 200 V for 30 min in a Mini‐Protean Tetra cell (Bio‐rad) using TGX running buffer (Bio‐rad). Gels were washed with distilled water and stained with Instant Blue (Expedeon) for 1 hr.

Gels were imaged in a Molecular Imager ChemiDoc XRS System (170‐8070, Bio‐rad) under white light epi‐illumination. Images were saved as a TIFF file with a size of 16‐bit and a resolution of 1,392 × 1,040 pixels.

### 2.4 ImageJ

Images were analyzed using ImageJ (NIH), a public domain program from the National Institutes of Health that allows image processing. Images were cropped to 1,070 × 774 pixels to zoom into the gel, equivalent to 0.1493 pixel/µm.

### 2.5 Brightness and contrast adjustments

Brightness and contrast adjustments were made using the automatic function of the program which oversaturates the pixels outside an automatically selected grayscale range. The adjustment is made using the histogram of the image.

### 2.6 Background subtraction

The function for background subtraction in ImageJ is based on the rolling ball algorithm of Sternberg (Sternberg, 1983). The algorithm simulates a spherical ball passing under the 3D profile of the optical density of the image. Increasing the size of the rolling ball radius decreases the background subtracted. This function is applied to the entire image. The rolling ball radius size was set to 50, 150, and 250 pixels for the analysis of data processing.

### 2.7 Oversaturation

For each analysis, the oversaturation of the maximal and minimal values of the grayscale was calculated from the histogram of gray values. For 16‐bit images, the histogram represents the results of 256 bins over the range of the grayscale values of the selected image. The oversaturation over black and white, maximal and minimal values, respectively, was made using the smallest and highest bin on the histogram of the entire image.

### 2.8 Densitometry

The Gel Analyzer tool of ImageJ was used to determine the profiles of each lane of the gels. The size of the lane selection tool was 16 pixels wide (Figure 2a), equivalent to 30% of the total width of the well as suggested by Gassmann et al. (2009). The lanes were always positioned at the center of the gel lane.

The profiles of the lanes were represented as the average of the grayscale values or the uncalibrated optical density along a one‐pixel‐height horizontal lane.

The calculation of the uncalibrated optical density uses an 8‐bit copy of the 16‐bit original image, and the grayscale values of the image are used according to Equation 2.

(2) $$\text{Uncalibrated}\;\text{OD}=\log_{10}\left(\frac{255}{\text{pixel}\;\text{value}}\right)$$

From the profile of the lanes, the area of the peaks was created manually, drawing a straight line across the baseline of the profile (Figure 2b).

## 3 RESULTS AND DISCUSSION

### 3.1 Optimal image processing with BSA as protein standard

With the objective of simultaneously determining the molecular weight and the protein load of the nanobody band on an SDS‐PAGE gel, the optimal image processing was identified using the BSA.

The oversaturation and the smoothness of the profiles were used as parameters for the selection of the optimal image modification. Of the seven tests performed, one was used as a control (automatic brightness and contrast adjustment and no background subtraction), and three different values of the background subtraction function (50, 150 and 250 pixels) were done using or not the automatic brightness and contrast adjustment. In Table 1, the tests performed to the raw image are displayed, with the results on the oversaturation on the maximal (over black) and minimal (over white) bin value.

**Table 1:** Tests performed for the image manipulation selection

Test on BSA	1	2	3	4	5	6	7
Brightness and contrast correction	Auto	Auto	Auto	N. A.	N. A.	N. A.	Auto
Rolling ball radius [pixel]	N. A.	50	150	50	150	250	250
Oversaturation over black [%]	0.19	0.00	0.04	0.00	0.00	0.00	0.00
Oversaturation over white [%]	0.01	20.05	10.8	18.34	10.33	6.56	6.29
Flattened peaks at high protein load	Yes	Yes	Yes	No	No	No	Yes
*R* ^2^ for grayscale profile [/]	.822	.852	.896	.935	.917	.872	.879
*p*‐Value for grayscale profile [/]	.013	.009	.004	.002	.003	.006	.006
*R* ^2^ for OD profile [/]	.993	.950	.974	.995	.992	.984	.975
*p*‐Value for OD profile [/]	2.28E−04	9.43E−04	2.54E−04	7.90E−06	2.44E−05	9.57E−05	2.29E−04

Note

Brightness and contrast adjustments were made with the automatic function. The background subtraction was made using the rolling ball radius at the given size. Flattened peaks found in both optical density and grayscale profiles. *R* ^2^ and p‐values were calculated for the BSA.

Abbreviation: N. A.: not applied.

The protein load was correlated with the peak area of the protein in the densitometry profile (Figure 2b). The peak area is the signal used in most densitometry analysis (Gassmann et al., 2009; Gorr & Vogel, 2015; Rehbein & Schwalbe, 2015), and the peak maximum intensity has been used in the quantification of proteins in Western blots imaged by fluorescence (Gürtler et al., 2013; Holzmüller & Kulozik, 2016). The volume of the peak is not commonly used (Vincent et al., 1997) since the correlations obtained often yield inaccurate protein load estimation (Gassmann et al., 2009).

The protein load (P, ng) in the well was calculated for each concentration (*C*, mg/ml) of the protein standard in the volume (V, mL) of 10 µl of sample diluted in Laemmli buffer (Equation 3).

(3) P=C∗V∗(1/2)

The results show that at high protein load, the grayscale and uncalibrated optical density profiles arrived at saturation levels when the brightness and contrast were adjusted. This led to profiles with plateaus at the top of the peaks in both profiles losing information of protein quantitation.

The oversaturation of white pixels increased when the radius of the background subtraction was reduced, according to the rolling ball algorithm. Conversely, the automatic correction of brightness and contrast decreased the oversaturation of white pixels between 5% and 10%. Oversaturation is a result of overprocessing the image that leads to a loss of information in the image (Cromey, 2010).

The grayscale profiles and the uncalibrated optical density profiles were compared by performing linear regression analysis between the BSA load and the peak area. The coefficients of determination and p‐values of each linear regression are shown in Table 1. The correlation of peak area with protein load using uncalibrated optical density profiles showed a better fit (*p*‐value < 1 × 10^–4^, *R* ^2^ > .97) than the ones of the grayscale intensity.

The optimal data treatment chosen was Test 6, with background subtraction at the size of the rolling ball of 250 pixels and no adjustment of brightness and contrast. This image processing protected the integrity of the data without losing important information. The uncalibrated optical density profiles were used for the linear regression analysis of the BSA, CA, and OV, and the correlations established had a good fit (*p*‐value < 1 × 10^–4^, *R* ^2^ > .98, Table 2).

**Table 2:** Regression analysis for bovine serum albumin (BSA), carbonic anhydrase (CA), and ovalbumin (OV) and molecular weight markers

Protein	BSA	CA	OV	100 kDa	50 kDa	20 kDa
Slope [(pixel*AU)/ng]	4.032	3.749	2.375	2.058	3.193	7.524
*σ* [(pixel*AU)/ng]	0.257	0.185	0.046	0.176	0.064	0.380
*R* ^2^ [/]	.984	.99	.998	.986	.999	.995
*p*‐Value [/]	9.57E−05	3.49E−05	8.62E−07	7.26E−03	3.98E−04	2.54E−03

Note

Equation was established as A = slope*P. A: peak area, P: protein load.

The difference in the slopes of the correlations could be attributed to the protein–Coomassie dye bond. The Coomassie stain binds primarily to basic amino acids such as arginine, histidine, and lysine, and to a lesser extent, to tryptophan and phenylalanine (Congdon, Muth, & Splittgerber, 1993; Stoscheck, 1990; Tal, Silberstein, & Nusser, 1985). The combined weight of these amino acids in the proteins is 22.41% mol/mol, 20.69% mol/mol, and 16.84% mol/mol for BSA, CA, and OV, respectively (https://www.uniprot.org, https://web.expasy.org/protparam). Unfortunately, a correlation between the molar content of those amino acids and molar slopes could not be found.

The fluorescence of tryptophan residues under UV‐light has been used for the quantification of proteins in SDS‐PAGE using Stain‐Free™ technology (Bio‐rad; Holzmüller & Kulozik, 2016). Protein quantitation was possible for proteins containing up to 5.25% mol/mol of relative tryptophan.

### 3.2 Quantification of molecular weight marker bands

According to the manufacturer (Bio‐rad), the ratio of intensities between the three reference bands (75, 50, and 25 kDa) and the seven other bands of the molecular weight marker should be 3, but it was not the case in this analysis. The ratio ranged from 2 to 3.4 using the maximum intensity of the peaks and from 4.8 to 17 using the peak area along the three molecular weight lanes (10, 5, and 1 µl, Figure 2a).

Using the information of protein load per band (Bio‐rad) for the bands of 100, 50, and 20 kDa, and the different loads in the SDS‐PAGE gel (10, 5, and 1 µl), the amount of protein per band was positively correlated with the peak area using Microsoft Excel 2010 (Microsoft Company; *p*‐value < .001, *R* ^2^ > .98, Table 2). Ranging from 2.058 to 7.524 pixel*AU/ng, the difference in the slopes of each molecular weight marker could be explained from their composition in amino acids. Unfortunately, this piece of information was not provided by the manufacturer (Bio‐rad).

The migration of the molecular weight marker on the gel could also explain the big difference in the slopes. The choice of gel, buffer, and running conditions of electrophoresis can modify slightly the migration of the bands. In the case of the Any kD Mini‐Protean TGX Stain‐Free™ Precast gels (Bio‐rad) used in these experiments, the gel gives a maximum resolution to proteins below 75 kDa, which is optimum for the nanobody CH10‐12 with a molecular weight of 31 kDa.

### 3.3 Quantification of an unknown protein

The quantification of an unknown protein could be made using the correlations established earlier for either the protein standards: BSA, CA, and OV, or the molecular weight markers: 100, 50, and 20 kDa. The choice depends on the type of molecule to be quantified, in this case, the nanobody CH10‐12 with a molecular weight of 31 kDa.

**Figure 3:**
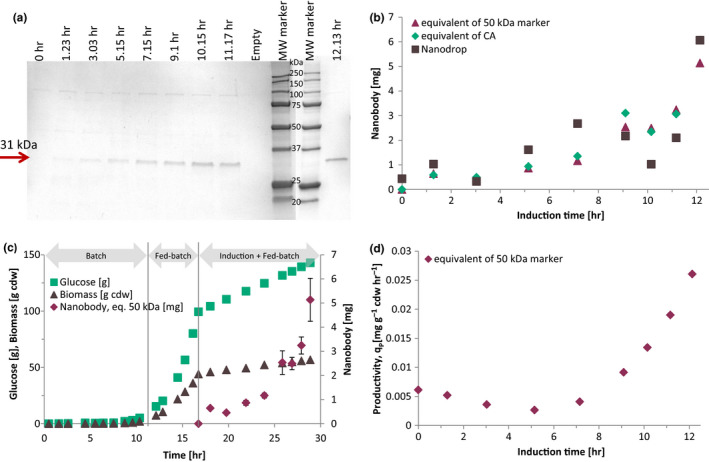
Quantification of a nanobody in SDS‐PAGE using densitometry. (a) Nanobody identification in SDS‐PAGE during protein expression. (b) Comparison between quantification using densitometry and nanodrop. (c) Nanobody production strategy during microbial culture of strain *E. coli* WK6/CH10‐12. (d) Productivity of the nanobody from ImageJ measures

The unknown concentration of a nanobody protein expression was obtained from 7 SDS‐PAGE gels stained with Instant Blue and analyzed by ImageJ with background subtraction (Figure 3a). The molecular weight marker was electrophoresed with the nanobody samples, and only the first two eluates were electrophoresed and quantified by densitometry. The concentrations of the purified nanobody were recalculated from the eluates of purification steps to the concentration in the culture (Figure 1).

The peak of 50 kDa in each gel was used as a reference for 750 ng, and linear regression with the intercept in zero was recalculated for the quantification of the nanobody. For comparative purposes, the correlation for CA was used to quantify the nanobody. The protein in the bands of the gel ranged from 33 to 258 ng per band for the molecular weight marker correlation and from 36 to 217 ng with the CA, corresponding respectively to concentration from 0.235 to 2.571 mg/L of culture and from 0.255 to 3.038 mg/L of culture.

The quantification of the protein bands using the CA correlation gives values on average 17% larger than using the correlation for the 50 kDa molecular weight marker. The use of one or the other can be used since the slopes are not significantly different.

The quantification by densitometry was compared with the nanodrop quantification of the samples (Figure 3b). The difference between the densitometry and nanodrop measures is due to the low concentration of protein in the samples, which falls in the lower limit of detection (0.1 mg/ml in the eluted fraction).

It has been reported a method of documentation and quantification of electrophoresis gel, using a series of diluted protein standards of known concentration electrophoresed with unknown samples (Rehbein & Schwalbe, 2015). The disadvantage is that at least one protein standard of known concentration must be used and electrophoresed with the samples in order to do the quantification.

In the present study, a protocol of optimum image processing in ImageJ was identified with the use of protein standards: BSA, CA, and OV. The linearity of the optical density profile versus protein load was tested in the protein standards and in the molecular weight markers. The molecular weight markers used in SDS‐PAGE were electrophoresed with the nanobody CH10‐12 samples and used to quantify the protein load, as well as to determine the molecular weight of the sample. This reduces the material costs related to the use of multiple protein standards and polyacrylamide gels.

### 3.4 Monitoring of nanobody production

The evolution of the periplasmic production of the nanobody CH10‐12 during protein expression in a fed‐batch bioreactor is represented in Figure 3c. During biomass production in the batch phase, the specific growth rate reached a maximum specific growth rate of 0.71 per hour. During the fed‐batch phase of biomass production, an average *µ* of 0.38 per hour was obtained with the exponential glucose feed. The nanobody was produced only after induction with IPTG (Figure 3c), and biomass growth was linear, with an average *µ* of 0.02 per hour.

The metabolic needs of the coupled production of the nanobody CH10‐12 and biomass were satisfied with the fed‐batch after induction, which has not been reported yet. The final titer of the nanobody CH10‐12 was 3.04 mg/L in a fed‐batch culture, which yield a total of 6.06 mg of nanobody CH10‐12. The maximum instantaneous productivity was obtained at the end of the culture with 0.026 mg/(g cdw*hr), and the average productivity was 0.0088 mg/(g cdw*hr; Figure 3d).

## 4 CONCLUDING REMARKS

This study has presented a protocol for image processing and protein quantification in electrophoresed gels. The densitometric analysis of protein standards of known concentrations (BSA, CA, and OV) and molecular weight marker bands (100, 50, and 20 kDa) was discussed for the quantification of unknown proteins in SDS‐PAGE gels. The protein band of 50 kDa in the molecular weight marker was used for the quantification of a nanobody during protein expression in a bioreactor.

The evolution of the periplasmic expression of the nanobody CH10‐12 was obtained in fed‐batch culture, with a maximum productivity of 0.026 mg/(g cdw*hr) after 12 hr of induction.

The optimum image processing was obtained by background subtraction with a rolling ball size of 250 pixels without brightness and contrast adjustment. The uncalibrated optical density was used for the correlation between protein load and peak area. The advantage of this methodology is the use of the molecular weight marker for the quantification of the protein in SDS‐PAGE gels without the use of additional protein standards.

## CONFLICT OF INTEREST

None declared.

## AUTHOR CONTRIBUTION

Susana María Alonso Villela: Conceptualization (equal); Formal analysis (equal); Methodology (equal); Writing‐original draft (equal); Writing‐review & editing (equal). Hazar Kraïem: Writing‐review & editing (equal). Balkiss Bouhaouala‐Zahar: Formal analysis (equal); Writing‐review & editing (equal). Carine Bideaux: Writing‐review & editing (equal). César Arturo Aceves Lara: Supervision (equal); Writing‐review & editing (equal). Luc Fillaudeau: Formal analysis (equal); Supervision (equal); Writing‐review & editing (equal). 

## ETHICS STATEMENT

None required.

## Data Availability

All data generated or analyzed during this study are included in this published article.
